# Combined Application of 17*β*-Estradiol and Progesterone Enhance Vascular Endothelial Growth Factor and Surfactant Protein Expression in Cultured Embryonic Lung Cells of Mice

**DOI:** 10.1155/2009/170491

**Published:** 2009-03-01

**Authors:** Andreas Trotter, Markus Kipp, Roland Matthias Schrader, Cordian Beyer

**Affiliations:** ^1^Section of Neonatology, Center for Perinatal Medicine, Children's Hospital, University of Bonn, 53105 Bonn, Germany; ^2^Institute of Neuroanatomy, RWTH Aachen University, 52057 Aachen, Germany

## Abstract

Preterm delivery is associated with disruption of the placental supply with 17*β*-estradiol (E2) and progesterone (P). The aim is to evaluate the role of E2 and P on the regulation of key proteins in lung development in embryonic lung cells. Alveolar cell type II (AT-II) and central lung fibroblast cultures were established from mouse embryos. Cells were exposed for 24 hours to E2 and/or P, the estrogen receptor antagonist ICI 182.780 (ICI) and the progesterone receptor antagonist mifepristone (RU 486). The mRNA expression of vascular endothelial growth factor (VEGF) and surfactant protein B and C (SB-B, SB-C) was determined, and protein levels of VEGF were measured. Only the combined treatment with E2 and P increased mRNA expression and VEGF protein in AT-II cells and lung fibroblasts. Combined treatment also promoted SP-B and SP-C expression in AT-II cells. Pretreatment with ICI and RU 486 completely abolished the E2 and P induced effects. E2 and P enhanced expression of VEGF and surfactant proteins in primary embryonic lung cells and may be involved in regulating expression of key molecules for the prenatal lung development and postnatal lung function.

## 1. Introduction

Respiratory distress syndrome (RDS) and bronchopulmonary dysplasia (BPD) remain major factors for morbidity and mortality in extremely preterm infants. Histopathological studies in preterm infants dying from BPD demonstrate an arrest of lung development with reduced alveologenesis [1]. The concept has evolved that postnatal lung development in extremely preterm infants is arrested by lack of factors that regulate lung differentiation and maturation in utero [2]. During mid and late gestation, the human fetus is exposed to high amounts of 17*β*-estradiol (E2) and progesterone (P) [3] produced by the placenta from precursors originating from the mother and the fetus [4]. Delivery disrupts the placental supply of both hormones. Within one day, the levels of E2 and P drop 100-fold [5]. This is a physiologic condition for the term infant. The extremely preterm infant is disrupted from the supply of these hormones at a much earlier developmental stage. The uterus as a known estrogen responsive target grows in utero until the end of gestation but stops growing in preterm infants [6]. It is conceivable that the withdrawal of E2 and P at this early developmental stage also affects lung development. Replacement of E2 and P in extremely preterm infants tailored to maintain in utero plasma levels of E2 and P was associated with a trend toward a reduced incidence of BPD [5, 7].

In mice lung mRNA expression of estrogen and progesterone receptors suggests that E2 and P are likely to be involved in mammalian fetal lung development [8]. The number of alveolar crests and alveolar type II cells [9] as well as lamellar bodies in type II cells [10] increased in rat fetuses after maternal E2 administration and E2 stimulates fetal lung surfactant production in rabbits [11, 12]. In newborn piglets antagonism of E2 and P during mid to late pregnancy decreased alveolarization [13].

Vascular endothelial growth factor (VEGF) is a major mitogen for vasculogenesis and angiogenesis [14] and is essential for embryonic development. Loss of a single VEGF allele results in embryonic lethality [15]. Absence of isoforms of VEGF impairs lung microvascular development and delays airspace maturation in mice fetuses reflecting the essential role of VEGF for normal lung development [16]. Pneumotrophic effects may be mediated through modulation of VEGF expression with the potential to accelerate lung maturation in preterm infants [17]. Intra-amniotic injection of VEGF in preterm rats resulted in increased surfactant protein B (SP-B) mRNA expression [18]. Type 2 pneumocytes respond to VEGF by increasing their expression of SP-B and SP-C [17].

Both sex steroids E2 and P and VEGF are described to induce surfactant proteins in the developing lung. Whereas it is established that E2 and P induce VEGF gene transcription in breast tumor cell lines [19] and the endometrium [20–22] no data from the literature is available about the effects of E2 and P on VEGF gene transcription in lung tissue. The aim of this study was to investigate if E2 and P are involved in the developmental regulation of VEGF and surfactant protein B (SB-B) and C (SB-C) mRNA expression using an in vitro model of cultured embryonic lung cells.

## 2. Material and Methods

All experiments followed the local guidelines according to the Federation of European Laboratory Animal Science Associations recommendations and were approved by local executives.

### 2.1. Cell Culturing and Treatment

Highly enriched alveolar cell type II (AT-II) and central lung fibroblast cultures were established from embryonic day (ED) 18 BALB/c mice as followed. Since E18 mouse lungs can be dissected from the surrounding vessels in a reliable manner we did choose this developmental stage to isolate purified AT-II cells from the mouse lung. Briefly, the fetuses were removed on ED 18 by caesarian section and the lungs aseptically explanted from the thorax. Lungs were then washed three times in 10 mL ice cold HBSS, mechanically dissected from surrounding vessels, and then incubated in a 2.5% trypsin solution containing 200 *μ*L of DNAse 1 (2 mg/mL, Worthington) for 10 minutes at 37°C in a shaking water bath (60 rpm). Finally, the cell suspension was filtered through a nylon mesh with 100-*μ*m pore size. The cell suspension was centrifuged twice at 420 and then 120 g for 4 minutes, and the pellet was resuspended in minimal essential medium (MEM). The resulting cell suspension contained the AT-II cells and attaching fibroblasts. After adding 1.5 ML collagenase (1250 U/mL, Worthington) and 150 *μ*L DNAse 1 (2 mg/mL, Worthington) the suspension was incubated for 15 minutes at 37°C. After stopping the collagenase activity by incubating in ice-cold MEM (supplemented with 10% FCS) the cell suspension was centrifuged as above for 4 minutes and the pellet was resuspended in MEM + 10% FCS. Cells were seeded and cultivated in a fibronectin-coated culture flask for 1 hour at 37°C under 5% CO_2_/21% O_2_. During this time, fibroblasts were attached to the flask, while AT-II cells did not due to their differential adherence characteristics. This procedure was repeated twice. Finally, approximately 1 × 10^5^ AT-II cells/cm^2^ were seeded at 24-well culture plates, cultured for one day in MEM supplemented with 10% FCS. Medium was changed after 24 hours to Cellgro complete medium (Mediatech, Virginia, USA, without phenol red). Attached fibroblasts were resuspended by trypsination and replated. When cells reached a confluence of approximately 80% the medium was changed and the final treatment was started. Cells were exposed for 24 hours to E2 and/or P and dexamethasone (all from Sigma-Aldrich, Germany) in concentrations ranging from 10–10 M to 10–6 M. Pretreatment with the estrogen receptor antagonist ICI 182,780 (TOCRIS bioscience, UK, 0.1 *μ*M) and the progesterone receptor antagonist mifepristone (RU 486 from Biomol, Germany, 0.1 *μ*M) was performed 1 hour before hormone exposure. Concentrations of ICI and RU 468 were derived by preparing a 10–2 M stock solution in ethanol 100% p.a. quality and by further diluting in medium. Appropriate ethanol concentrations served as controls.

### 2.2. Gene Expression Analysis

The mRNA expression of VEGF, SP-B, and SP-C was quantified using the rtPCR technology (BioRad, Germany), QTM SYBR Green Supermix (BioRad, Germany), and a standardized protocol as described previously [23, 24]. Isolation of total RNA was performed with peq Gold (PeqLab, Germany). RNA concentration and purity were assessed using OD260 and OD260/OD280 ratio, respectively, and reverse transcribed using an Invitrogen M-MLV RT-kit and random hexanucleotide primers. The rtPCR reactions were carried out in a reaction mixture consisting of 2 *μ*L cDNA, 6 *μ*L RNAse-free water, 10 *μ*L hot StartTaq DNA-polymerase, and 1 *μ*L of primer (10 pmol). Reactions were conducted in standard tubes using the MyIQ rtPCR Detection System (BioRad, Germany) under following conditions: 10-minute enzyme activation at 95°C, 45 cycles of 15-second denaturation at 95°C, 30-second annealing at individual temperatures, 30-second amplification at 72°C, and 5-second fluorescence measurement at 80°C. Primer sequences to detect VEGF, SP-B, and SP-C are shown in Table 1. Relative quantification was performed using the Δ*Ct* method, which results in ratios between target genes and a housekeeping reference gene (HPRT). As the validity of this method critically depends on the constant expression of the housekeeping gene, constant expression of HPRT was tested against other housekeeping genes (not shown). In each run, external standard curves were generated by several fold dilutions of target genes. The concentration of the target genes was calculated by comparing *Ct* values in each sample with *Ct* values of the internal standard curve. Finally, data were expressed as the ratio of the amount of each transcript versus the concentration of HPRT. Melting curves and gel electrophoresis of the PCR products were routinely performed to determine the specificity of the PCR reaction.

### 2.3. Protein Analysis

An ELISA for VEGF was conducted according to the manufacturer's instructions (RayBiotech ELISA Kit, specific for VEGF-A). For each sample, blank values (i.e., those for serum-free media) were subtracted, and mean results were normalized per 10^5^ cells, counted after trypsination in a Neubauer's counting chamber. This assay was performed in triplicate experiments.

### 2.4. Immunostaining

Purity of cell cultures was determined by immunohistochemistry for vimentin (fibroblasts, abcam, Germany, 1:100) and cytokeratin (AT-II cells, abcam, Germany, 1:75). Cells were grown on top of autoclaved 13 mm circular glass cover slips placed in each well of a 24-well plate. After incubation, the media were aspirated, and cells were washed twice in PBS. Cells were fixed in methanol and incubated with 1% BSA and 2% FCS in PBS for 20 minutes at 23°C prior to exposure to primary antibodies for 18 hours at 4°C. Appropriate secondary antibodies and the AEC Kit (Zymed Laboratories, Calif, USA) were used for staining.

### 2.5. Statistical Analysis

Differences between groups were tested by one-way analysis of variance (ANOVA) followed by Tukey's post hoc multiple range test using SPSS software (SPSS Inc., Chicago, IL, USA). A *P*-value of less than .05 was considered significant. Results in figures are presented as means with standard error of the mean.

## 3. Results

### 3.1. Cell Culture Purity and Receptor Expression

**Figure 1:**
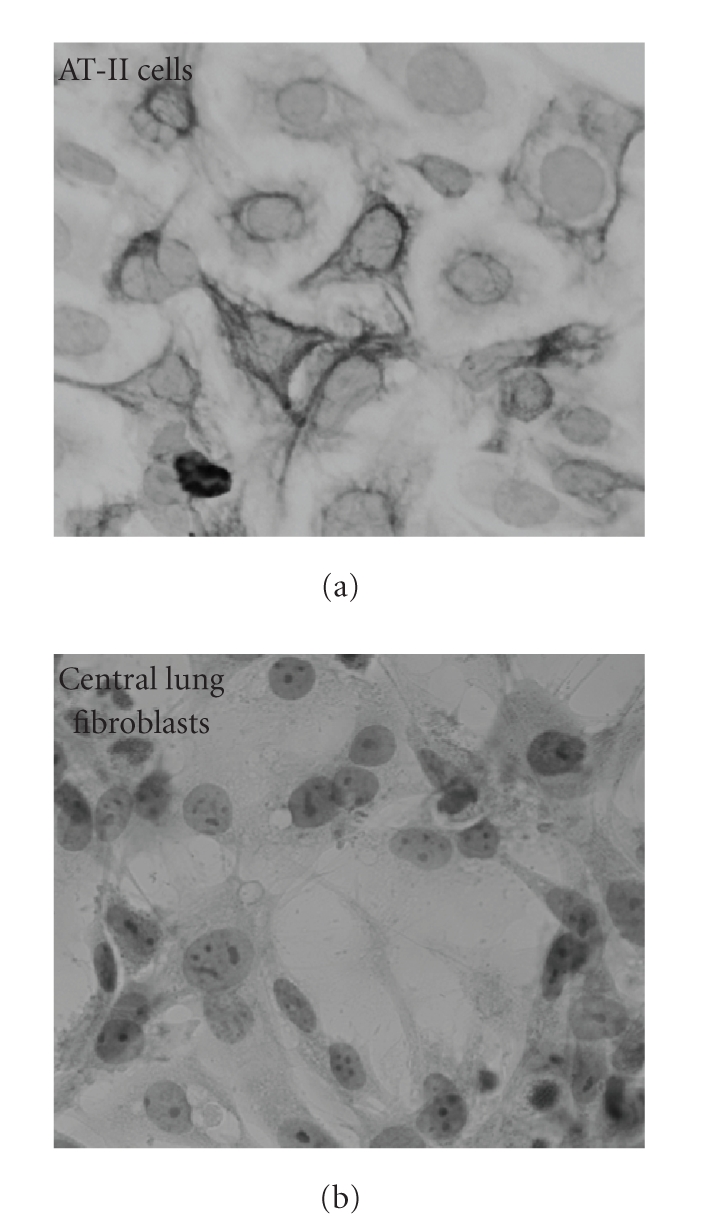
Alevolar cells type II (AT-II) and central lung fibroblasts stained with cytokeratin antibody. Note that AT-II stain positive for cytokeratin whereas fibroblasts do not. Therefore, contamination of AT-II cells in fibroblast cell cultures can be excluded.

**Figure 2:**
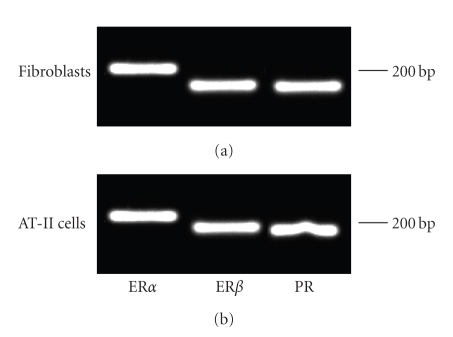
Semiquantitative analysis of estrogen receptor alpha (ER-*α*), estrogen receptor beta (ER-*β*), and the progesterone receptor (PR) expression in central lung fibroblast and AT-II cell cultures. Note that all three hormone receptors are expressed in both cell cultures.

The culturing protocol for lung fibroblast yielded a greater than 98% homogeneity of vimentin-positive fibroblasts. The culturing protocol for AT-II cells yielded a greater than 95% homogeneity of cytokeratin-positive cells. Cross contamination of purified fibroblasts cell cultures was excluded by immunohistochemical staining of fibroblast cell cultures with anti-cytokeratin antibody (Figure 1). No expression of SP-B and SP-C was detected in lung fibroblasts further proving purity of cell cultures. Semiquantitative PCR revealed that all three hormone receptors, namely, estrogen receptor alpha (ER-*α*), estrogen receptor beta (ER-*β*), and the progesterone receptor (PR) are expressed in purified central lung fibroblast and AT-II cell cultures (Figure 2).

### 3.2. Hormonal Effects on Central Lung Fibroblasts

**Figure 3:**
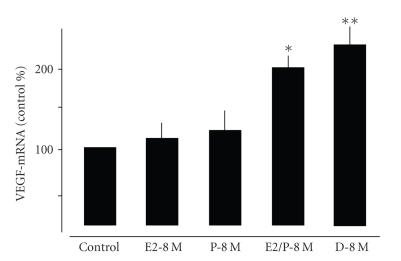
Quantitative analysis of VEGF gene expression in central lung fibroblasts treated for 48 hours with E2-8 M and P-8 M alone or in combination. Values were normalized against a housekeeping gene (HPRT) and expressed as % of controls. Note that only the combined application of both hormones significantly increased VEGF expression in central lung fibroblasts. Also note that the application of dexamethasone (D) had similar effects on VEGF expression. *P < .01 control versus E2/P-8 M, **P < .01 control versus D-8 M.

**Figure 4:**
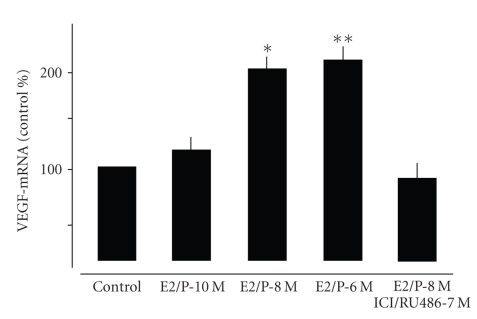
Quantitative analysis of VEGF gene expression in central lung fibroblasts treated for 48 hours with increasing concentrations of both E2 and P and with ICI/RU 486. Values were normalized against a housekeeping gene (HPRT) and expressed as % of controls. Note that the application of receptor antagonists 1 hour prior to hormone application (ICI/RU 486) antagonizes hormonal effects. *P* < .01 control versus E2/P-8 M; **P < .01 control versus E/P-6 M.

**Figure 5:**
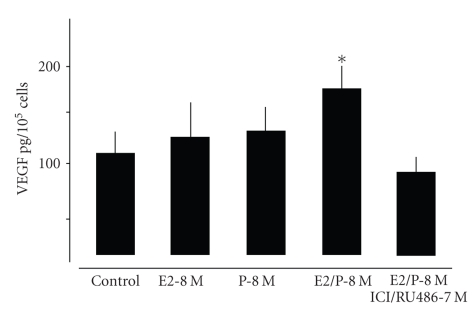
Quantification of VEGF protein release of lung fibroblasts treated for 48 hours with E2-8 M and P-8 M alone or in combination determined by ELISA. Note that corresponding with results obtained by gene expression analysis (Figure 3) VEGF protein is increased in central lung fibroblast cultures only by combined treatment with E2 and P. Pretreatment with ICI/RU 486 abrogated this effect. *P < .05 control versus E2/P-8 M.

The treatment of cultured fibroblast with either E2-8 M or P-8 M alone for 48 hours had no significant influence on VEGF mRNA expression (Figure 3). However, a combined application with both steroids resulted in a significant increase by approximately 100% compared to controls in VEGF mRNA levels (*P* < .01, Figure 3). Dexamethasone was applied as positive control (*P* < .01, Figure 3). The combined application at concentrations lower than 10–8 M did not significantly affect VEGF mRNA expression (Figure 4), and higher concentrations (10–6 M) did not further promote VEGF mRNA expression compared to 10–8 M (Figure 4). The hormone-induced upregulation of VEGF mRNA was completely blocked by the application of the receptor antagonists ICI and RU 486 (Figure 4). The single or combined treatment with ICI and/or RU 486 did not influence the basal expression of investigated proteins as determined by rt-PCR analysis (data not shown). Using ELISA analysis, we could confirm the transcriptional regulation of VEGF by E2 and P. Only the combined application increased extracellular VEGF protein levels in fibroblasts (Figure 5). Pretreatment with the receptor antagonists again abrogated this effect.

### 3.3. Hormonal Effects on AT-II cells

**Figure 6:**
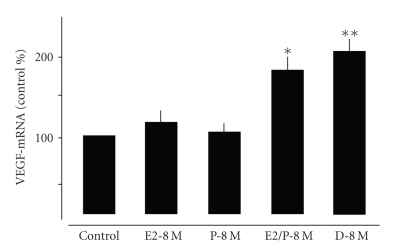
Quantitative analysis of VEGF gene expression in alveolar cells type II treated for 48 hours with E2-8 M and P-8 M alone or in combination. Values were normalized against a housekeeping gene (HPRT) and expressed as % of controls. Note that only the combined application of both hormones significantly increased VEGF expression in central lung fibroblasts. Also note that the application on dexamethasone had similar effects on VEGF expression. *P < .01 control versus E2/P-8 M, **P < .01 control versus D-8 M.

**Figure 7:**
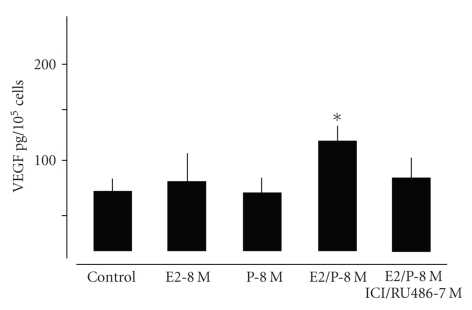
Quantification of VEGF protein release of alveolar cells type II treated for 48 h with E2-8 M and P-8 M alone or in combination determined by ELISA. Note that corresponding with results obtained by gene expression analysis (Figure 6) VEGF protein is increased in alveolar type II cell cultures only by combined treatment with E2 and P. Pretreatment with ICI/RU 486 abrogated this effect. *P < .05 control versus E2/P-8 M.

**Figure 8:**
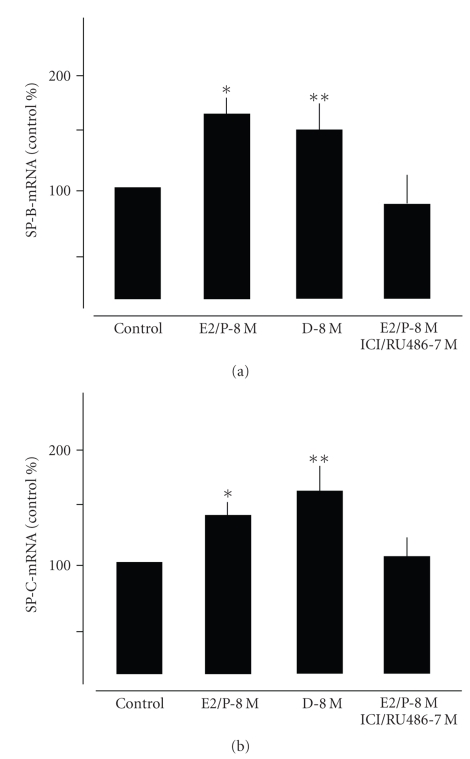
Quantitative analysis of SP-B and SP-C gene expression in alveolar cells type II treated for 48 hours with E2-8 M/P-8 M in combination or with dexamethasone-8 M. Values were normalized against a housekeeping gene (HPRT) and expressed as % of controls. Note that combined hormonal treatment increased expression of both surfactant proteins to a similar extent like dexamethasone. Further note that pretreatment with the receptor antagonists ICI and RU 486 antagonized the hormonal effects. *P < .01 control versus E2/P-8 M; **P < .01 control versus D-8 M.

As shown for fibroblasts only the simultaneous exposure to E2-8 M and P-8 M significantly enhanced the expression of VEGF (Figure 6) and this could be confirmed at the transcriptional level (Figure 7). Dexamethasone also increased VEGF amount in AT-II cells (Figure 6). Combined application of E2 and P increased mRNA expression of SP-B and SP-C to a similar extent as dexamethasone (Figure 8). Pretreatment with the receptor antagonists ICI and RU 468 abrogated this effect. SP-A was not found in AT-II cells, however was expressed in mature lung tissue which did serve as a positive control (not shown).

## 4. Discussion

The role of angiogenic growth factors for developmental processes is increasingly recognized [25]. A decreased expression may be a potential mechanism of alveolar capillary dysmorphogenesis in BPD. In animal models of bronchopulmonary dysplasia and in preterm infants dying from BPD, diminished VEGF mRNA expression is evident (for a review see [25]). VEGF stimulates the growth of lung epithelial cells in vitro [26] and is important for pulmonary vascular development [27]. Mice with a deficiency of VEGF die from RDS [17], and VEGF knockout results in embryonic lethality [15]. To our knowledge no data on the influence of E2 and P on VEGF expression in the developing lung is available. In primary lung fibroblasts and AT-II cells only the combined application of E2 and P resulted in increased expression levels of VEGF mRNA and VEGF protein. This effect was abolished by pretreatment with the specific E2 and P antagonists ICI and RU 486, respectively. Assuming that E2 and P increase VEGF expression in the developing lung in vivo, the withdrawal of the placental supply of E2 and P in preterm infants may explain the disturbed lung development and function. Postnatal replacement of E2 and P in preterm infants was associated with a trend toward reduced incidence of BPD [5, 7].

Disturbed lung function in RDS of preterm infants is due to surfactant deficiency. A regulative role of E2 in surfactant synthesis is supported by enhanced mRNA expression for SP-B found in fetal rabbit lung cells [28]. Our data supports a regulative role of E2 and P for surfactant synthesis as in AT-II cells combined treatment with E2 and P resulted not only in increased VEGF but also in increased mRNA expression of SP-B and SP-C. We did not perform experiments to evaluate the role of VEGF for surfactant synthesis. However, data from the literature implicates a trigger function of VEGF for surfactant synthesis. Intra-amniotic injection of VEGF in preterm rats resulted in increased SP-B mRNA expression [18]. Furthermore, type II pneumocytes respond to VEGF by enhancing their expression of SP-B and SP-C [17]. We speculate that E2 and P promote VEGF production and, thereby, surfactant synthesis.

One major finding of our study is that only the combination of E2 and P was effective in the upregulation of VEGF, SP-B, and SP-C expression. This is in accordance with findings about the epithelial Na^+^-channel which plays a critical role in the active reabsorption of alveolar fluid at the time of birth and during pulmonary oedema. In rats, only the combined application of E2 and P promoted mRNA levels of the epithelial Na^+^-channel in the lung suggesting complex interactions between the intracellular E2 and P signalling [29]. It appeared that only the administration of both hormones is fully effective in preventing demyelination in a multiple sclerosis animal model [30]. It is well known that ER and PR are coexpressed in the same cells in several areas of the target tissues [31, 32]. In addition, a number of reports demonstrated that ER and PR can have synergistic or inhibitory cross-talk in their transcriptional regulation in promoter type- and PR subtype-specific manners [33, 34]. Also interactions on well-known nongenomic levels are assumed. For example, in breast cancer cells, estrogens activate the Src/Erk pathway through an interaction of the ER with the SH2 domain of c-Src. Progestins have been reported to activate also this pathway either via an interaction of the PR with ER, which itself activates c-Src, or by direct interaction of PR with the SH3 domain of c-Src [35]. Future studies have to show the underlying mechanisms for combined positive hormonal effects. The preterm infant is deprived of both E2 and P, simultaneously. Our study adds evidence that only combined replacement of E2 and P may be effective to prevent BPD in preterm infants [5, 7].

Treatment with dexamethasone increased VEGF mRNA expression and VEGF protein in lung fibroblasts and AT-II cells. In contrast, in vitro studies have shown that dexamethasone downregulates VEGF expression in cells derived from alveolar epithelial cells [36]. However, in mid-trimester fetal human lung explants dexamethasone increased VEGF mRNA expression [37] and this at least at the translational level was also found in vivo [38]. Interestingly, treatment of preterm infants with dexamethasone was associated with increased VEGF levels in deep pulmonary lavages [39].

The embryos were not stratified by gender. Therefore, we cannot exclude that by chance some experiments were conducted in cell cultures from predominant male or female embryos. The steroid concentrations being most effective in our experiments, that is, 10–8 M, appear at first glance to be above the physiological plasma levels found in rodents during the estrous cycle. However, it is generally accepted that under in vitro conditions higher steroid levels are required to yield cellular effects. In previous in vitro studies, these high estrogen concentrations were applied to generate physiological effects [40, 41]. Another more intriguing point is that tissues themselves produce steroids. Thus, tissue intrinsically synthesized steroids may contribute together with plasma steroids to reach higher local tissue steroid concentrations. In support for this view is the recent observation that in rodent hippocampal tissue local estrogen production yields tissue steroid levels at approximately 10–9 M which are greater than those in the plasma [42].

In conclusion, combined use of E2 and P enhanced expression of VEGF and surfactant proteins in primary embryonic lung cells. These proteins are known to be key factors for the prenatal lung development and postnatal lung function. Further research about the effects of E2 and P on lung development may open therapeutic perspectives for preterm infant prone to develop lung disease.

## Figures and Tables

**Table 1:** Primer sequences for mRNA detection of the different gene products.

Gen	Forward	Reverse	bp	AT
VEGF*	cca cgt cag aga gca aca tca	tca ttc tct cta tgt gct ggc ttt	71	60
SP-B	cca cct cct cac aaa gat gac	ttg ggg tta atc tgg ctc tgg	174	60
SP-C	atg gac atg agt agc aaa gag gt	cac gat gag aag gcg ttt gag	119	60
ER-a	cgt gtg caa tga cta tgc ctc	ttt cat cat gcc cacttcgtaa	199	62
ER-b	ctg tga tga act acagtg ttc cc	gca gtg ggt ggc taa agg a	124	62
PR	cca act tca caa aac ttc tcg aca	ggc agc aat aac ttc aga cat ca	127	64
HPRT	gct ggt gaa aaggac ctc t	cac agg act aga aca cct gc	248	62

*recognizes transcript variants VEGF 120, 144, and 164.

## Abbreviations and Conversion Factors

AT-II: Alveolar cells type II
BPD: Bronchopulmonary dysplasia
D: Dexamethasone
E2: 17*β*-Estradiol, pg/mL × 3.671 = pmol/L
ER-*α*: Estrogen receptor alpha
ER-*β*: Estrogen receptor beta
ICI: ICI 182.780 = 7*α*-[9-[(4,4,5,5,5-Pentafluoropentyl)sulphinyl]nonyl]-estra-1,3,5(10)-triene-3,17 *β*-diol
M: Molar
MEM: Minimum essential medium
Mrna: Messenger ribonucleic acid
P: Progesterone, ng/mL × 3.18 = nmol/l
PR: Progesterone receptor
RDS: Respiratory distress syndrome
RU 486: Mifepristone
SP-B: Surfactant protein B
SP-C: Surfactant protein C
VEGF: Vascular endothelial growth factor A
